# Lyme Disease and Other Tick-Borne Infections

**DOI:** 10.1007/s11910-026-01530-1

**Published:** 2026-10-20

**Authors:** John J. Halperin, Gary P. Wormser

**Affiliations:** 1 Department of Neurology, Atlantic Health Overlook Medical Center, 99 Beauvoir Avenue, Summit, NJ 07901 USA; 2 Sidney Kimmel Medical College of Thomas Jefferson University, Philadelphia, PA USA; 3 Division of Infectious Diseases, Department of Medicine, New York Medical College, Valhalla, NY 10595 USA

**Keywords:** Tick, Lyme disease, Powassan virus, Tick-borne encephalitis virus, *Ehrlichia chaffeensis*, Anaplasma, Babesiosis, *Borrelia miyamotoi*

## Abstract

**Purpose of Review:**

The neurologic manifestations of selected tick-transmitted infections in North America and Eurasia were reviewed.

**Recent Findings:**

Both Powassan virus in North America, and Tick-Borne Encephalitis (TBE) virus in Eurasia can cause encephalitis, often with significant neurologic morbidity. Lyme disease, *Ehrlichia chaffeensis* (the cause of human monocytic ehrlichiosis [HME]), and *Borrelia miyamotoi* infections can cause meningitis (the third primarily in immunocompromised individuals). Patients with Lyme disease may also develop cranial neuropathy, and/or radiculoneuropathy.

**Summary:**

Tick-borne infections are being increasingly recognized in North America and Eurasia. While many tick-borne infections can cause headaches, lethargy or other non-specific symptoms, these are generally not evidence of nervous system infection – i.e., neuroinvasive disease. The latter is less common but may occur in infections due to Powassan and TBE viruses, in HME and in Lyme disease*.* Patients with symptomatic nervous system infection due to Powassan or TBE virus are at high risk of developing significant neurologic impairment and sequelae.

## Introduction

Recent years have seen a substantial increase in reported cases of certain tick-borne infections in North America. While Lyme disease (LD) is the best known of these, others are receiving increasing attention. This review will consider the potential neurologic manifestations of selected tick-borne infections.

For most of the discussed infections, *Ixodes* species ticks serve as the vector *– I. scapularis* in the Northeast and Midwest regions of North America, *I. pacificus* in the western US, *I. ricinus* in parts of Europe and *I. persulcatus* in parts of Eurasia. These infections are zoonoses – infections transmitted to humans from other animal species – for these, transmission is by a tick bite. All have a competent reservoir animal host, such as field mice or other small mammals, occasionally larger animals or birds, which typically carry the pathogen asymptomatically. After pathogen ingestion by a tick feeding on the reservoir host, pathogens must be able to survive in the tick, remaining viable for a sufficient period of time, and then potentially being transmitted, by a subsequent tick bite, to a second animal host, such as a human. Historically, much of the focus on this process has involved infections caused by *Borrelia burgdorferi* sensu lato* (Bbsl)* – the spirochetal agents which cause LD. However, *Ixodes species* ticks are also potential vectors for other human pathogens, including *Anaplasma phagocytophilum* (the cause of human granulocytic anaplasmosis [HGA]), *Babesia microti* (the most common cause of babesiosis in the US), and both Powassan and Eurasian tick-borne encephalitis (TBE) viruses. *Borrelia miyamotoi* – a relapsing fever borrelia (~ 40 US cases/year [1]) – will be considered only briefly – because of its rarity, Two other *I. scapularis* transmitted pathogens – *Ehrlichia muris eauclairensis* (~ 20 US cases/year) and *Borrelia mayonii* which may cause a LD-like illness (< 10 US cases diagnosed through 2023 [2]) will not be discussed. Finally, although Bartonella*,* the cause of cat scratch disease, is considered by some a potential LD co-infection, there is no evidence that Bartonella is transmitted to humans by ticks in North America [3], so this infection will not be considered here.

Another important pathogen – included in this review because of its potential for nervous system involvement—is *Ehrlichia chaffeenis,* the cause of human monocytic ehrlichiosis (HME). Unlike the other pathogens mentioned above, *E. chaffeensis* is transmitted by the lone star tick, *Amblyomma americanum,* the tick whose bite is responsible for alpha-gal syndrome [4]*.*

*Ixodes* species-transmitted infections largely follow similar seasonality, with the risk of infection highest during the summer but extending from spring through autumn. Besides North America, *Ixodes-*transmitted infections occur in Scandinavia, in temperate central Europe, and in other parts of Eurasia. Generally, the tick distribution is more widespread than that of the pathogens they carry, reflecting the required co-localization of infected reservoir hosts.

Just as tick-borne pathogens vary in the animal hosts they can infect, they also vary in their organotropism within infected hosts. The blood–brain and blood-cerebrospinal fluid (CSF) barriers protect the central nervous system (CNS) from most infections. However, some pathogens have developed the ability to breech these barriers. In assessing the reported frequency of neurologic involvement for a particular pathogen, 4 factors must be kept in mind. First, for many infections, such as for flaviviruses including Powassan and TBE, many if not most infections are either asymptomatic or cause only mild non-specific symptoms and consequently are never specifically diagnosed. Consequently, acute nervous system infections may dominate reported cases, creating the false impression that most infected patients have a devastating neurologic disease. Second, in many non-neurologic infections systemic inflammation results in a non-specific toxic-metabolic encephalopathy, which should not be construed as evidence of CNS infection. Third, headaches occur in many infections but only rarely are due to intracranial infection, so diagnosing nervous system infection based solely on the occurrence of headaches is inappropriate. Finally, particularly in uncommon infections, single case reports of purported neurologic complications may simply be coincidental and not causally related. That said, Table 1 lists the number of cases reported annually to the CDC – numbers that are an under-estimate of actual case counts but likely approximate the relative frequencies of the various infections—and the frequency of nervous system infection reported for each.

**Table 1:** Annual number of confirmed US cases of selected tick-borne infections per the CDC. Note that CDC case reporting uses strict case definitions, chosen to assure specificity, recognizing that this may reduce sensitivity. For example, current estimates of overall US Lyme disease incidence is approximately 500,000 cases/year [5], as opposed to the 89,468 cases meeting the updated CDC surveillance criteria in 2023. For the other listed pathogens the number of infected, but untested individuals with no or only non-specific symptoms is indeterminate – for Powassan virus in particular, the diagnosis is typically only considered in patients with otherwise unexplained encephalitis

	US Cases/yr	Reported % with CNS involvement	Year of data
Lyme disease	89,468	12–15%	2023
Babesiosis	3,586	Rare?ever	2023
A. phagocytophilum	7,280	Very rare/never	2023
E. chaffeenis	1,907	Uncommon	2023
Powassan virus [6]	60	90%	2025
B. miyamotoi	~ 40	Infrequently; primarily in immunocompromised	2023

### Lyme Disease

LD, or Lyme borreliosis (LB), is the most prevalent tick-borne human infection in North America. Given its propensity for nervous system involvement, it should not be surprising that it is also the most frequent tick-borne infection to affect the nervous system. As with other *Ixodes*-transmitted infections, its geographic distribution in the US, while expanding [7] remains relatively localized with 90 + % of reported cases from 15 states: Connecticut, Delaware, Maine, Maryland, Massachusetts, Minnesota, New Hampshire, New Jersey, New York, Pennsylvania, Rhode Island, Vermont, Virginia, West Virginia, and Wisconsin [8]. While LB is widespread in Europe, infections tend to cluster in southern Scandinavia and temperate central Europe.

In comparing European and North American LD it is usually stated that neurologic involvement is more common in European patients, with arthritis more frequent in US patients – differences attributed to the specific organotropisms of the predominant Lyme *Borrelia species* in these different locations. While it is clear that arthritis is significantly more common with the predominant US strain, *B. burgdorferi* sensu stricto* (Bbss*), data regarding nervous system involvement is less clearcut. European infections are most often caused by *B. afzelii* and *B. garinii, B. garinii* being particularly associated with nervous system infection*.* Overall, in Europe Lyme neuroborreliosis (LNB) is said to affect up to 12% of LB patients [9, 10] while US CDC data [11] describe nervous system involvement in 12.5%.

Although these percentages are remarkably similar, European and US data are not directly comparable. The European Federation of Neurologic Societies (EFNS) criteria for diagnosis of definite or possible neuroborreliosis require specific CSF findings [12] that are not required by CDC criteria [13] or by US Guidelines [14]. Without this requirement in the US, cases not meeting more rigorous criteria are likely included, increasing the apparent number of US LNB cases. At the same time, the overall number of CDC-reported LB cases is thought to be a substantial underestimate of the actual number [5]. Assuming diagnosed LNB cases would be reported more consistently than erythema migrans (EM), these factors might together artificially increase the apparent proportion of LNB cases in the US. At the same time, European case reporting varies from country to country, suggesting that the number of reported cases in Europe might also lack precision.

The other plausible explanation for perceived differences in nervous system involvement is historical. Lyme disease [15] was originally identified as a form of arthritis – a much less prominent element of European LB – with *Bbss* having a greater propensity to cause large joint oligo-arthritis than *B. afzelii* or *B. garinii*. LNB in the US was only recognized as an additional manifestation of the already established entity, Lyme arthritis, several years after its first characterization [16]. In Europe LNB constituted – and remains—the first described and principal extra-cutaneous manifestation – a source of clinical anchoring bias.

Central to these considerations is the diagnostic approach to these infections. Typical EM skin lesions, when seen in an appropriate epidemiologic setting, are sufficiently characteristic to diagnose and treat LD without further testing. This is important as serologic testing is insensitive early in infection, being negative in 50% or more of EM patients in the US [17, 18]. For other manifestations of LD, however, diagnosis in the US relies on 2-tiered serologic testing [14].

EFNS criteria for the diagnosis of definite or probable CNS LNB require CSF assessment, with determination of the Lyme antibody index (AbI) – the ratio of the proportions of *B. burgdorferi-* specific antibody in CSF and serum—to establish the presence of intrathecal production of specific antibody to *B. burgdorferi*. Only patients with a positive AbI, and a CSF pleocytosis, can be diagnosed with definite LNB. Those with just 1 of the 2 are considered to have possible LNB. Sensitivity of the AbI is generally considered to be about 90% [12, 19–21]. Notably, like peripheral blood serologies, the AbI can remain positive for years after successful treatment [22], an important limitation of this indicator.

While there are several technical approaches to determining the AbI, all require simultaneous CSF and serum samples, then measurement of CSF and serum specific antibody after normalizing for total immunoglobulin of the class being assessed. This can be performed by a capture assay, or by measuring CSF and serum IgG and/or IgM, then either diluting CSF and serum to the same final immunoglobulin concentration and assaying, or by assaying the 2 fluids at standard dilutions, then titrating the results and correcting arithmetically [23].

Importantly, both CSF nucleic acid testing and CSF culture have very low diagnostic sensitivity, and national guidelines recommend against performing either [14].Oral doxycycline provides effective treatment for most patients with LNB – with IV ceftriaxone or other IV antibiotics reserved for individuals with the most severe involvement. In the US, LNB diagnosis is often made clinically, without CSF examination, once the diagnosis of LD itself has been established.

Neurologic abnormalities in European patients were first described decades before the responsible organism was identified. A 1922 case report by Garin and Bujadoux [24] is usually cited as the first published example. Despite inaccuracies pointed out a century later [25], the case description is remarkable. A 58-year-old sheep herder was bitten on the left buttock by a tick, developed an enlarging erythematous skin lesion lasting weeks to months, and developed severe neuropathic pain in a bilateral sciatic nerve distribution, then a right brachial plexopathy, with symptoms persisting for months. CSF demonstrated a pleocytosis with a slightly positive Wasserman reaginic test for syphilis. The authors concluded this was an infection caused by a non-syphilitic spirochete, treated the patient with neoarsphenamine (a treatment for neurosyphilis at the time), and the patient rapidly recovered. Meningitis and severe painful radiculopathy remain 2 of the 3 key elements of LNB in Europe – to which cranial neuropathies, most often the facial nerve, were subsequently added.

European studies generally identify painful radiculoneuropathy as the most common presentation in adults with LNB (referred to as Garin-Bujadoux-Bannwarth syndrome) occurring in up to 86% of individuals with neurologic findings [26–28] – only 20% of whom have weakness in the affected anatomic distribution. Cranial neuropathies occur alone, or in combination with radicular symptoms, in just over one-half, with most cranial neuropathies involving the facial nerve – one-third of which are bilateral. Headaches occur in less than one-half of cases, and meningeal signs in under one-quarter of cases despite the presence of a CSF lymphocytic/monocytic pleocytosis in most.

Of the 12.5% of LB patients identified by the US CDC as developing LNB, 2/3 had facial palsy, 30% painful radiculoneuropathy, 10% isolated lymphocytic meningitis and < 0.1% encephalitis (i.e., inflammation of the brain parenchyma) [11], differing from European patients primarily in the relatively lower frequency of radicular symptoms. While anecdotal observations suggest that this may be due to under recognition, this has not been studied systematically. In most other ways, findings in US patients with peripheral nerve involvement are very similar to those in Europeans. In both, CSF may be abnormal but often is not – not surprising for clinical manifestations limited to peripheral nerve [29]. Radiculoneuropathy symptoms often are more sensory than motor, but both can be prominent. Localization can be to 1 or several nerve roots, to a plexus, to single or multiple nerves in a somewhat patchy distribution. In all, the neurophysiologic pattern is most consistent with a mononeuropathy or mononeuropathy multiplex, consistent with the findings from the few available nerve biopsies – which have shown patchy axon loss and perivascular inflammatory infiltrates [29].

European studies describe a different pattern in children, in whom facial nerve palsy (which is otherwise quite uncommon in children) is by far the most common presentation [30]. Although most have a CSF pleocytosis, only a minority have meningeal signs. Although one study described radicular symptoms in about one-half of children with LNB [26], these were much milder than in adults. Recent reviews of LD in US children similarly identify facial nerve palsy as the most common neurologic manifestation, affecting 5% of all pediatric LD patients [31], whereas radicular symptoms are reported rarely, with no incidence data provided [32]. Of the few available studies, one [33] found neurologic findings in 32 of 96 children with LD—13 with facial nerve palsy; none with radicular symptoms. US CDC data [11] describe an increased incidence of neurologic involvement in children ages 5–14 with LD but do not break this down by clinical presentation. In a Canadian study of 170 children presenting to one Emergency Department, 13 (8%) had facial nerve palsy; none had painful radicular symptoms [34]. A population-based analysis of Canadian children with LD additionally described “other neurologic manifestations” in 14.5% – identified only as “e.g., meningitis” but with no mention of radicular symptoms [35]. Overall, it appears that in children with LD in both Europe and North America, painful radiculitis is extremely uncommon.

A late cutaneous manifestation seen only in European patients, acrodermatitis chronica atrophicans, is usually caused by *B. afzelii*. Skin in the affected limb becomes thin, wrinkled and reddish-blue. Up to one-half of affected patients develop predominantly sensory symptoms [36], most frequent and most severe in the clinically affected limb [36–39]. Diminished sensation is often but not invariably seen in symptomatic areas. Neurophysiologic findings are consistent with a disseminated axonal polyneuropathy [36–38]. Additional studies, including detailed neurophysiologic testing would be helpful to better characterize this.

With regard to CNS involvement, in both North American and European patients, lymphocytic/monocytic meningitis is commonplace – with highly variable symptoms, ranging from being asymptomatic to having severe headaches with neck stiffness, independent of the degree of inflammation as reflected by the CSF cell count. Parenchymal CNS involvement is quite rare. Spinal cord involvement at the symptomatic root level in Garin-Bujadoux-Bannwarth syndrome occurs but probably less commonly in US than European patients. Children, in whom symptoms are often milder than adults, may develop a picture resembling idiopathic intracranial hypertension [40] – except that it is usually associated with, or follows, Lyme meningitis. While patients generally recover fully, those who have focal neurologic deficits as part of the acute illness may have neurologic residua of these deficits. Beyond these residua, a substantial number of patients describe cognitive and memory symptoms after what is typically microbiologically curative antibiotic treatment. Controlled studies suggest that in adults [41] this occurs only slightly more often (< 4%) than in control populations, in children [42] at the same frequency as controls, with available evidence indicating that this is unrelated to continued infection or inflammation.

One aspect that has been the source of considerable controversy over the years has been the entity referred to as “Lyme encephalopathy”. While patients actively ill with LD, similar to patients with other ongoing inflammatory states, may perceive cognitive and memory difficulty, evidence does not suggest this reflects CNS infection or inflammation [43]. Early suggestions of a possible association of these symptoms with what are now described as “non-specific white matter abnormalities” on brain MRI have been discarded based on improved and more systematic neuro-imaging [44].

Finally, and importantly, the best available evidence – ranging from case control to large population studies—does not support previously suggested associations between LD and a myriad of other neurologic disorders, with population studies failing to demonstrate epidemiologic associations between LD and Alzheimer’s disease, amyotrophic lateral sclerosis, Parkinson’s disease or multiple sclerosis in the US [45] or of LB with any of these 4 entities, or with epilepsy, Guillain Barre syndrome [46], psychiatric disorders [47] or pediatric attention deficit disorder, learning or developmental disorders [48] in Denmark.

### Borrelia miyamotoi

Despite its very low reported incidence (approximately 40 cases/year in the US), *B. miyamotoi* infection is frequently considered [1]. Importantly, it is a member of the relapsing fever group of *Borrelia*, a group not otherwise considered in this review because of their quite limited neuro-invasiveness. *B. miyamotoi* infection commonly causes headaches, myalgias and arthralgias but like other relapsing fever borrelia*,* is infrequently neuro-invasive. Diagnostic testing often relies on serologic testing but this has limited specificity [1, 49, 50], with low sensitivity acutely, increasing to 78% on convalescent phase serum samples [49]. Diagnosis is made most reliably with whole blood PCR. Specificity issues notwithstanding, seroprevalence studies have been performed [51] suggesting that 2.8% of a New England population had been exposed to *B. miyamotoi.* Nervous system infection is quite uncommon—typically symptomatic chronic meningitis [52] with a CSF pleocytosis with symptoms of confusion but otherwise non-focal neurologic assessments– occurring primarily in immunocompromised individuals [53] in whom parenchymal CNS involvement can occur as well. However this is very uncommon in immunocompetent individuals [54].

### Babesiosis

Although there are multiple babesia species, most infections in the US are caused by *B. microti*, with smaller numbers caused by *B. divergens* and *B. duncani* [55], while in Europe *B. divergens* and *B. venatorum* predominate. Nervous system infection is unusual, with rare case reports of coma or focal findings [56], although the specificity of these relationships is unclear. In one series of 34 hospitalized patients in a highly endemic area [57], 24% developed headaches, 26% myalgias, 9% mental status changes, but none had evidence of nervous system infection. A more recent study [58] identified 163 adults hospitalized with babesiosis at Yale-New Haven Hospital over a decade. Headache occurred in 32%, confusion/delirium or impaired consciousness in 31%, gait difficulty in 10%, with altered mental status associated with higher levels of parasitemia. Six percent of patients described transient visual impairment but had normal ophthalmologic examinations. Four died; in 2 of these this was preceded by altered mental status. Of the 33 (20%) who underwent brain imaging, 2 had abnormalities observed – an ischemic stroke in a patient with severe internal carotid stenosis and a subarachnoid hemorrhage in a patient with disseminated intravascular coagulation. CSF was examined in 6 patients and was essentially normal in all. As this series illustrates, neurologic symptoms can appear due to complications of systemic involvement, but there appears to be little evidence that babesiosis is directly neuro-invasive.

### Anaplasmosis

*Anaplasma phagocytophilum* is a gram-negative obligate intracellular bacterium that infects granulocytes, causing HGA. Infections may be asymptomatic or may cause an acute febrile illness, with severity increasing with older age and comorbidities. CNS involvement is very rare.

A recent systematic review [59] found that overall, fever was present in 90% of symptomatic patients, malaise in 59%, myalgias in 42%, headache in 37%, confusion in 8%, with 55% describing GI symptoms ranging from anorexia to vomiting and diarrhea. However, there was no consistent pattern of nervous system involvement, and CSF examinations were usually normal, regardless of symptomatology [60]. A recent review of 120 patients diagnosed with HGA in Europe [61], many with co-infections, described headaches in 56%, meningeal signs in 22% but no specific CNS abnormalities. CSF findings were summarized in patients with TBE co-infection but otherwise not described. Isolated reports [59] have described seizures, stroke-like symptoms and peripheral nerve involvement. One report [62] described a patient with lethargy, headache and confusion, felt to have aphasia but no other focal abnormalities. Brain MRI showed leptomeningeal enhancement and blood over the left frontal lobe but no parenchymal abnormalities. MR angiogram (day 2) demonstrated middle cerebral artery irregularity, thought to be indicative of post-subarachnoid hemorrhage vasospasm – notwithstanding that this is rarely seen before days 3 or 4 after a bleed. Head CT on day 3 did not demonstrate intracranial bleeding, and CSF was clear and colorless (no xanthochromia) and normal except for 1,460 RBCs with 6 nucleated cells. Extensive testing for other potential etiologies was negative, although there was no testing for Powassan virus. Although this patient had a positive blood anaplasma PCR, the relationship between this infection and the neurobehavioral symptoms is unclear. Another case report described a patient who presented with low grade fever, mild headache, and mild confusion but was otherwise neurologically normal – who was thought to have meningoencephalitis [63]. CSF showed a mild pleocytosis but was otherwise normal, as was a contrast enhanced brain MRI; this patient recovered without sequelae. Given the absence of any demonstrable CNS damage by neurologic examination or brain MRI, the evidence seems insufficient to conclude that this patient had encephalitis. Another case report attributed a small lacunar infarct to HGA [64]. However, the case description and MRI images are more consistent with this lacune being an incidental asymptomatic and non-acute finding, unrelated to the acute illness. Overall, the anecdotal nature of these isolated case reports and lack of consistent CSF or brain MRI abnormalities makes it difficult to attribute any CNS involvement to HGA with certainty. There appears to be agreement that HGA is highly unlikely to cause neurologic disease or sequelae, with no evidence clearly demonstrating that this microorganism can cause a chronic infection [60].

### Ehrlichiosis

*Ehrlichia* [65, 66], like *anaplasma*, are gram negative obligate intracellular bacteria, transmitted to humans by tick bites, with their geographic distribution reflecting that of the transmitting ticks. The most common, *Ehrlichia chaffeensis*, which causes HME, is transmitted to humans by the bite of lone star ticks—*Amblyomma americanum*—originally most common in Missouri, Oklahoma and Arkansas, now spreading through much of the south central and southeast US, and north along the east coast. The principal reservoir host is white tail deer. Once infected during feeding on a reservoir host, the tick carries the pathogen through its life cycle. In humans, the bacteria live within phagosomes in monocytes (morulae), which can be visualized by light microscopy after Giemsa staining, although PCR testing of whole blood samples has much higher diagnostic sensitivity.

While most patients with HME have only non-specific symptoms and recover spontaneously or with doxycycline, in some the course is more complex. In one of the earliest detailed clinical descriptions, [67] 40 patients, presumably infected with *E. chaffeenis*, were identified retrospectively serologically; 34 had been hospitalized. All reported fever, headache, chills, malaise, nausea, myalgia and anorexia. Ten had a maculopapular rash, 5 a petechial rash. Fourteen patients had pulmonary infiltrates on chest Xray; 7 required mechanical ventilation; and 4 developed acute renal failure. Six had altered mental status with 3 progressing to coma. CSF was examined in 14; 10 had a lymphocyte-predominant CSF pleocytosis, and 8 had increased CSF protein. Two with underlying medical problems died. While all of these studies incorporate a selection bias in that only a subset of patients underwent CSF examination, the available evidence supports there being low-grade meningeal inflammation in some patients with *E. chaffeensis* infections, but CNS parenchymal invasion or damage appears to be quite rare.

### Powassan and Tick-borne Encephalitis (TBE) Viruses

TBE virus infection [68] is endemic in central Europe – particularly in Austria, the Baltic states, the Czech Republic, Germany, southern Scandinavia and Slovenia, with about 4,000 cases reported in Europe annually – despite the availability of an effective vaccine—with the endemic area extending eastward through much of Asia. This enveloped, positive-sense, single-stranded RNA flavivirus, may be spread by *Ixodes* tick bites. About 2% of TBE infections are likely due to ingestion of infected dairy products, however, and not from a tick bite.

About 2/3 of infected TBE patients are asymptomatic. Among symptomatic patients, initial symptoms are non-specific, with fever, malaise, arthralgias and headache, typically subsiding, with the patients then becoming largely asymptomatic. About one week later, however, 2/3 will then develop a second phase of the illness. In 1 large registry study [69] of 555 patients who developed the second phase, this consisted of meningitis in 207, meningoencephalitis in 273, and spinal cord involvement in 74. Overall, 7% required ICU care, 2% died, and 59% had persisting neurologic sequelae. Common neurologic manifestations included altered consciousness (24%), ataxia or tremor (37%), cranial neuropathies (5%) and seizures (1%). Patients with encephalitis were the oldest in the group, those with meningitis, with or without spinal cord involvement, the youngest. Not surprisingly those with meningitis alone were most likely to recover (60%), those with encephalitis being the least (19%). Vaccination was highly effective for preventing infection –only 16 of the 555 cases had been vaccinated; 9 of these16 had received an incomplete vaccine schedule.

Powassan virus is a flavivirus closely related to the TBE virus, similarly transmitted by Ixodes species ticks. Unlike most of the tick-transmitted pathogens discussed here, but like TBE virus [70] and *B. miyamotoi* [71, 72]. it can spread from adult female ticks transovarially to their eggs. Diagnosis is typically serologic, particularly based on IgM seropositivity, but nucleic acid testing of CSF is available. Between 2006–2016, 99 cases of infection with this enveloped, positive stranded RNA flavivirus were reported to the US CDC [73] – 89 of these were neuro-invasive. Peak Powassan virus incidence was in May and June. Unlike other tick-borne infections impacting humans, transmission can occur within minutes of a tick bite [74], in contrast to the at least 36 h of tick attachment required for transmission of *B. burgdorferi* in North America. Most cases have been reported from eastern Canada, and northeast and north central US. There are 2 closely related lineages of the virus -lineage 1 and lineage 2. Although this was previously emphasized, it now appears that the lineages are so similar that this is no longer a clinically relevant distinction.

While case reporting is based on highly symptomatic patients, there have been several informative, although small, serosurveys. In one of patients and healthy controls in a LD endemic area [75], 9 of 95 suspected of having a tick-borne disease and 2 of 50 healthy controls were seropositive for antibodies to Powassan virus, suggesting that asymptomatic or minimally symptomatic infection occurs, probably more frequently than neuro-invasive disease [76]. Notably, blood from 1 of the Powassan virus seropositive patients suspected of having a tick-borne disease was also PCR positive. A second study of 52 patients with EM failed to identify any with serologic evidence of exposure to Powassan virus [77]. Whether this difference is due to small sample sizes or to geographic differences remains to be determined.

A review of cases reported to the CDC found that diagnosed infections most often present as encephalitis (72%), less often meningitis (16%), or as a non-specific febrile illness in 10% [73], numbers clearly skewed by testing being largely limited to individuals presenting with neuro-invasive disease. Overall mortality was 11%—all of whom were over 50 years old with neuro-invasive disease. A case series of 16 patients seen at the Mayo Clinic [78] had similar findings: 4/16 had meningitis, 12/16 parenchymal CNS involvement; and 3/16 died. Eight of the 13 survivors had neurologic sequelae. Results of a systematic review of published cases were comparable [79] with a mortality rate of 19% in adults and 7% in children; 44% of adults and 43% of children had motor neurologic residua; 33% of adults and 25% of children had residual cognitive difficulty. Ataxia, paralysis and speech difficulty occurring during the acute illness were particularly predictive of having residual neurologic deficits. Mortality and residual signs and/or symptoms all increased with increasing age. In 1 autopsied case [80], an early brain MRI demonstrated FLAIR hyperintensities in the cerebellum (which is particularly commonly involved in this infection [81]), pons and bilateral basal ganglia, with a subsequent brain MRI showing additional involvement of the thalamus and cerebral hemispheres. CSF very early in infection was acellular with minimal protein elevation; follow up CSF testing showed a lymphocyte predominant pleocytosis, an elevated protein level of 192 mg/dl and a normal glucose level– findings comparable to those reported in a systematic review [79]. At autopsy there was a widespread necrotizing meningoencephalitis with intra-neuronal virus identified by immunohistochemistry throughout much of the neuraxis.

### Conclusions

Of the tick-borne pathogens (see Table 2) discussed in this review, at least 4 have the potential for nervous system infection. Powassan and TBE virus may cause encephalitis, with significant potential for CNS damage and sequelae. *E. chaffeensis* can cause altered mental status and meningitis but rarely with long-term morbidity. Lyme disease causes all, or a part, of a triad of meningitis, cranial neuritis and radiculoneuritis, and very rarely encephalomyelitis; long-term neurologic morbidity can occur in Lyme disease patients who have neurologic deficits acutely but is otherwise quite uncommon.

**Table 2:** Symptoms with or without neuro-invasion, diagnostic tools and suggested treatment

Pathogen	Non-neuroinvasive manifestation	Neuroinvasive manifestation	Diagnostic test	Treatment (Adult)	Strength of evidence
*B. burgdorferi*	Headache, cognitive symptoms, fatigue	Cranial or radiculoneuropathy, meningeal signs	2 tier serologic testing (North America); CSF – cells, protein, CSF:serum Ab index, Opening pressure in children	Doxycycline – 200 mg/d po; 2–3 weeks Severe or parenchymal disease: IV ceftriaxone 2gm/d; penicillin 18-24MU/d, or cefotaxime 2 gm TID; 2–3 weeks	strong
*B. miyamotoi*	Headache, myalgias and arthralgias	Meningitis; encephalitis in immunocompromised	PCR; serology low sensitivity acutely & specificity limited	As above [52, 82] Ceftriaxone, 4 weeks in immunocompromised	By inference Anecdotal
A. *phagocytophilum*	Fever, chills, headache, and myalgias		Intragranulocytic inclusions on blood smear; PCR on blood; acute & convalescent serology	Doxycycline – 200 mg/d po; 10 days	moderate
Babesia [55]	Fever, chills, myalgia, arthralgia, anorexia, nausea, fatigue; headache, confusion/delirium, and impaired consciousness		Intra-RBC inclusion on blood smear; PCR on blood	Atovaquone + azithromycin or clindamycin + quinine; 7–10 d in immunocompetent patients	strong
*E. chaffeenis*	Fever, chills, headache, and myalgias	Meningitis; very rarely encephalitis	Inclusions on blood smear, PCR acute & convalescent serology	Doxycycline – 200 mg/d po; 10 days	moderate
*Powassan/TBE*	Fever, chills, headache,	Meningoencephalitis, ataxia	Serology (IgM); PCR on CSF MRI brain	supportive	strong

## Key References

- Lantos PM, Rumbaugh J, Bockenstedt LK, et al. Clinical Practice Guidelines by the Infectious Diseases Society of America (IDSA), American Academy of Neurology (AAN), and American College of Rheumatology (ACR): 2020 Guidelines for the Prevention, Diagnosis and Treatment of Lyme Disease. Clin Infect Dis 2021;72:1–8.
  - ○ Definitive review of evidence regarding Lyme disease diagnosis and treatment.
- Taylor-Salmon E, Shapiro ED. Tick-borne infections in children in North America. Curr Opin Pediatr 2024;36:156–163.
  - ○ Review of data on tick-borne infections in children.
- Locke S, O'Bryan J, Zubair AS, et al. Neurologic Complications of Babesiosis, United States, 2011–2021. Emerg Infect Dis 2023;29:1127–1135.
  - ○ Data from large case series of patients with babesiosis.
- Mendoza MA, Hass RM, Vaillant J, et al. Powassan Virus Encephalitis: A Tertiary Center Experience. Clin Infect Dis 2024;78:80–89.
  - ○ Summary of Mayo Clinic experience with Powassan encephalitis.

## Data Availability

No datasets were generated or analysed during the current study.
